# Associations of behavioral, motivational, and socioeconomic factors with BMI among children and adolescents

**DOI:** 10.1038/s41390-025-03860-1

**Published:** 2025-01-17

**Authors:** Charlotte Jungehuelsing, Christof Meigen, Sarah Krause, Wieland Kiess, Tanja Poulain

**Affiliations:** 1 LIFE - Leipzig Research Center for Civilization Diseases, Leipzig University, Philipp-Rosenthal-Strasse 27, 04103 Leipzig, Germany; 2 Department of Women and Child Health, Hospital for Children and Adolescents and Center for Pediatric Research (CPL), Leipzig University, Liebigstrasse 20a, 04103 Leipzig, Germany

## Abstract

**Background:**

Higher weight represents a significant health concern in youth and may be influenced by socioeconomic and behavioral factors. We investigated the relationship between BMI and parental education, nutritional health, eating culture, organized and non-organized physical activity (PA), motives for PA (weight loss/maintenance, enjoyment), and screen-time in children and adolescents.

**Methods:**

677 2- to 11-year-olds (young-age-group) and 464 12- to 20-year-olds (old-age-group) from Leipzig, a city in Germany, participated. We applied multivariate linear regression analyses to assess associations.

**Results:**

BMI-SDS was negatively associated with parental education (young-age-group: b = −0.25, *p* < 0.001, old-age-group: b = −0.27, *p* = 0.02), non-organized PA (young-age-group: b = −0.23, *p* = 0.029), and PA enjoyment (young-age-group: b = −0.05, *p* = 0.01, old-age-group: b = −0.05, *p* = 0.038), but negatively with media use during dinner (old-age-group: b = 0.53, *p* < 0.001), PA for weight loss/maintenance (young-age-group: b = 0.15, *p* < 0.001, old-age-group: b = 0.12, *p* < 0.001), and screen-time (young-age-group: b = 0.11, *p* = 0.009, old-age-group: b = 0.09, *p* = 0.001). Significant interactions with age, sex and parental education were observed.

**Conclusion:**

A lower BMI in children is associated with high parental education, screen-free eating, higher participation in non-organized PA and lower screen-time. While measures of motivation were limited and thus findings should be interpreted with caution, intrinsic motivation for PA is associated with lower BMI whereas extrinsic motivation for PA is associated with higher BMI.

**Impact:**

- In a German cohort of children and adolescents, lower BMI is associated with high parental education, less screen time, more participation in non-organized physical activity and less media use during dinner.
- Intrinsic and extrinsic motives for physical activity are directly linked to the weight status of children and adolescents.
- These associations are particularly strong in families with low/medium formal education.

## Introduction

In the last decades, the prevalence of childhood obesity has been shown to increase significantly.^1^ After the strong increase, prevalence rates have plateaued at a high level in many high income countries.^2^ In the “German Health Interview and Examination Survey for Children and Adolescents” (KiGGS) conducted between 2014 and 2017 in Germany, the prevalence of overweight and obesity among children and adolescents was 9.5% and 5.9%, respectively.^3^ During the COVID-19 pandemic, an increased weight gain among children and adolescents has been documented.^4^

This is worrying because childhood obesity represents a significant health concern. It is associated with depression, metabolic, cardiovascular and oncologic diseases in adulthood^5–7^ and may lead to premature mortality.^8^ An early onset predicts obesity in adulthood^9^ and is associated with more severe consequences^10^: Higher BMI levels at late childhood (5 to 8 years) are associated with increased risk of cardiometabolic diseases such as coronary artery disease, myocardial infarction and chronic kidney disease.^11^ Childhood BMI trajectories were shown to have a significant effect on adult diabetes, independent of BMI levels, and the adolescence age period is a crucial window for the development of adult diabetes.^12^

The development and maintenance of child and adolescent obesity can be explained by the socio-ecological model. This model suggests that different factors, acting at the individual, interpersonal, organizational, community and policy level, influence children’s health-related behavior and, therefore, their weight.^13,14^ Exampled for these factors and the resulting health behaviors are socioeconomic status (SES), diet, physical activity (PA) and sedentary behavior including media use.^15^ Children from families with a lower SES have a higher BMI.^16,17^ Furthermore, obesity rates are higher among children and adolescents with a lower SES.^18^

An unhealthy diet may increase the risk of obesity^19^ and eating habits also seem to play a role: Having the TV on while eating is associated with a poorer diet quality among children.^20,21^ Some studies have found that having the TV on during dinner is associated with a higher BMI,^20^ while others have found no such effect.^22,23^ Frequent family meals have also been shown to have a protective effect on healthy weight in children and adolescents.^24,25^ Snacking on highly processed foods and foods that are high in salt, fat or sugar is associated with increased weight gain in children.^26–28^ In addition, helping to prepare meals has been shown to be associated with a healthier diet.^29^ However it is unclear whether there is an association between meal preparation and weight status.^29^

Prospective studies further showed that PA is associated with lower rates of weight gain and obesity in childhood and adolescence.^30–32^ Little is known, however, on how motivational aspects of PA are related to children’s weight status. According to Ryan and Deci, intrinsically motivated actions are performed because they are inherently pleasurable or interesting, while extrinsically motivated actions are performed for a clear separable outcome.^33^ Appearance motives like weight control can therefore be considered extrinsic motives, while PA enjoyment can be regarded as intrinsic motive.^34^ PA enjoyment is related to PA engagement in adolescents^35,36^ and reduces the age-related decline in PA-levels.^37^ Preventing this decline and promoting motivational factors could play an important role in the development of exercise habits that persist in childhood and adolescence.^38^ Although studies have shown that young women with higher BMI tend to be extrinsically motivated to exercise (e.g., for weight loss),^39^ the connection between intrinsic and extrinsic motivation and children’s weight status requires further investigation.

The use of screen media has increased considerably among children and adolescents in recent years.^40^ To prevent excessive media consumption, a number of guidelines have been established, giving recommendations for daily maximum screen time.^41,42^ The link between extended screen time and higher BMI in children and adolescents is well documented^43,44^ and could be due to mindless eating in the absence of hunger^44^ as well as eating unhealthy foods while watching screens.^20^

The aim of the present study was to investigate the relationship between BMI-SDS (BMI Standard Deviation Score) and parental education (as indicator of SES), diet (nutritional health and culture of eating (media use during dinner, dinner with family, snacking, help cooking)), PA (participation in and duration of organized and non-organized PA), motives for PA (PA enjoyment and PA for weight loss/maintenance), and total screen time in a large study population. Based on previous findings, we expected BMI-SDS to be negatively associated with high parental education, nutritional health, dinner with family, help cooking, participation in and duration of organized and non-organized PA, and PA enjoyment. We expected a positive association between BMI-SDS and media use during dinner, snacking, PA for weight loss/maintenance and total screen time.

Finally, we expected the associations between BMI-SDS and the independent variables to be stronger in children and adolescents from families with lower SES.

## Methods

### Participants

The data analyzed for the current study were collected between 2021 and 2023 within the LIFE Child study. LIFE Child is a longitudinal childhood cohort study located in Leipzig, Germany, that investigates healthy development and the development of non-communicable diseases in children and adolescents.^45,46^ Since 2011, participants not suffering from any chronic, chromosomal, or syndromal diseases have been recruited between the prenatal period (24th week of pregnancy) and 16 years of age. Participants are invited to attend annual follow-up visits. They are recruited via advertisement at health institutions and schools, media and word of mouth.^45^ Most participants stem from the city or the proximity of Leipzig, a large city in the East of Germany. Leipzig is part of the Central German Metropolitan Region.

Parents gave informed written consent before inclusion of their children in the study. The LIFE Child study was designed in accordance with the Declaration of Helsinki and approved by the Ethics Committee of the Medical Faculty of Leipzig University (Reg. No. 477/19‐ek).

**Fig. 1:**
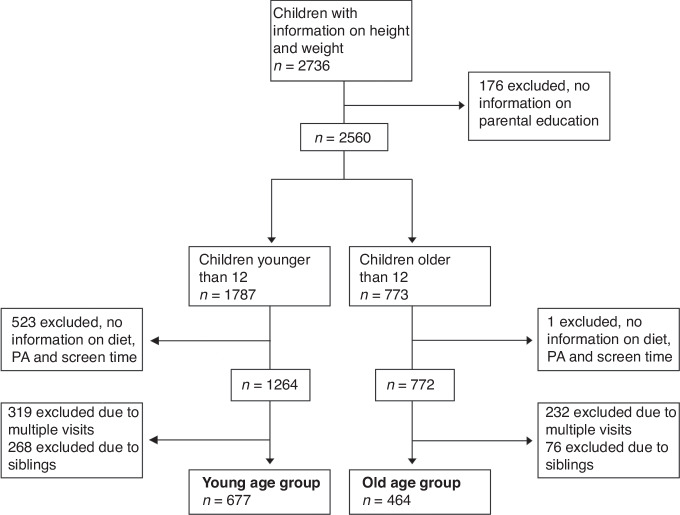
Flow diagram of inclusion an exclusion criteria.

All children who participated between 2021 and 2023 and for whom information on BMI, SES, diet, PA and screen time was available were eligible for the present analyses (see Fig. 1).

Analyses were stratified by age group. For children younger than 12 years (young age group), questionnaires about diet, PA and screen time were completed by their parents. Older children (old age group) completed these questionnaires themselves. In both age groups, parents gave information about their SES. Only the oldest sibling was included if siblings participated in LIFE Child. For children attending multiple times, only the first visit was selected in the young age group, and the last visit was selected in the old age group for a more balanced age distribution. In total, 677 children (47.7% girls, mean age = 7.71 years, range = 2–11) were included in the young age group and 464 children and adolescents (49,6% girls, mean age = 15.31 years, range = 12–20) were included in the old age group.

### Measures

#### BMI-SDS

Height (without shoes) and weight (in underwear) were measured by certified study assistants and converted into BMI values (kg/m^2^). The original BMI values were transformed into BMI Standard Deviation Scores (BMI-SDS) based on German sex- and age-specific reference values.^47^

#### Parental education

Parental education, as indicator of a family’s SES, was assessed by asking parents for their highest general school-leaving qualification as well as their highest professional qualification (for response options: see Supplement, Table 1). The responses were combined to a score ranging from 1 to 7, with higher values indicating a higher level of education, as described in Lampert et al. (2018).^48^ For data analysis, the education score was categorized as indicating either low/medium (1-6) or high education (7). For participants with missing information on maternal education, paternal education was treated accordingly and used in the analyses.

#### Diet

Dietary habits were analyzed using the Composition and Culture of Eating questionnaire (CoCu^49^), a screening instrument that assesses the composition and culture of eating in children and adolescents. The questionnaire consists of two parts: In the first part, participants/parents were asked how many portions of different food products they/their children consume per day (for fruits/vegetables, unsweetened milk products, sweetened milk products, sweetened beverages, wholegrain bread, white bread) or per week (for meat, fish, ready-made meals, fried potatoes, potatoes, rice/noodles, cakes, sweet or savory snacks). The answering options included 6 possible frequencies (1 = “never”; 2 = “max. 1”; 3 = “2 to 3”; 4 = “4 to 5”; 5 = “6 to 7”; and 6 = “ > 7 portions”). Descriptions of reference portions were given in the text (e.g. “a fistful”) or provided as photographs. The selection of items was based on the food groups included in a Food Frequency Questionnaire (FFQ).^50^

Based on the responses, the consumption of each food group was categorized as either healthy (10 points), moderately healthy (0 points) or unhealthy (−10 points). The scores for each food group were added up to create a nutritional health score (NHS) that ranges from −120 points (unhealthy) to 120 points (healthy). The scoring is based on the German dietary guidelines for children and adolescents,^51^ with specific parameters set for the level of consumption of each food product.

The second part of the questionnaire assesses culture of eating. We examined the questions “Do you/ does your child use the TV, tablet, smartphone, mobile phone or similar devices during dinner at home?”, “Do you/ does your child usually have dinner with your family?”, “Do you/ does your child usually snack between meals (e.g. chocolate, jelly babies, crisps, pretzel sticks)?”, and “Do you help your parents with the cooking?/ Does your child help with the cooking?”. Response options were binary, i.e., “yes” or “no”. Validity and reliability of the questionnaire were tested and confirmed in a previous study.^49^

#### Physical activity (PA)

The questions regarding PA were developed by researchers from LIFE Child. Study participants/their parents were asked to indicate whether they/their children currently engage in organized PA (e.g., in a sports club) and non-organized PA (e.g., self-organized) outside the school context. Both items were binary, the answer options were “yes” or “no”. We further asked how many hours per week they/their children engage in organized and non-organized PA. Participants/parents could answer both questions in 0.5 h increments. If children were not physically active (organized or non-organized PA = “no”), the time was considered as 0 h. In the young age group, the information on the duration of non-organized PA was missing in 261 children. Therefore, this analysis included fewer children.

Additionally, motives for PA were surveyed. Participants/parents were asked to indicate to what extent they/their children enjoy sports and PA in their daily lives, and to what extent they/their children engage in PA for weight loss or weight maintenance. The 11 response options ranged from 0 = “does not apply at all” to 10 = “applies completely”.

#### Media use

As for PA, the questions on media use were designed by the LIFE Child research team. For the assessment of total screen time, participants/parents were asked to indicate how many hours a day they/their children spend using screen media (TV, smartphone, PC, tablet, etc.), excluding media use for schoolwork, on weekdays and weekend days, respectively. For both questions, 26 answer categories were provided (“never”, “30 min a day”, “an hour a day”, […], “12 h a day”, and “more than 12 h a day”). After transformation of the responses to minutes per day, a composite score was calculated ((screen time on weekdays x 5 + screen time on weekend day x 2)/7).

### Statistical analysis

All statistical analyses were conducted using R (version 4.1.3). Descriptive statistics were reported as means and standard deviations for continuous variables and counts and percentages for discrete variables.

To evaluate possible associations between BMI-SDS and parental education, we performed a linear regression analysis with BMI-SDS as dependent variable and parental education as independent variable. The association was adjusted for sex and age, e.g., these variables were included as covariates.

To assess the associations between BMI-SDS (dependent variable) and the NHS, media use during dinner, having dinner with the family, snacking and help cooking, participation in and duration of organized and non-organized PA, motives for PA, and total screen time (independent variables), we performed a linear regression analysis for each independent variable, adjusting for age, sex, and parental education. In a second step, all characteristics that showed significant associations in these analyses were included in multivariate analyses.

Following our hypotheses, associations between BMI-SDS and all independent variables were checked for interactions with sex, age, and parental education. Effects were reported as non-standardized regression coefficients and the significance level was set to $\alpha$ = 0.05.

## Results

### Study population

The characteristics of the study population can be seen in Table 1. The distributions are presented separately for both study samples.

**Table 1:** Socio-demographic characteristics and characteristics of diet, culture of eating, PA, and total screen time in the young and the old age group.

		young age group (2–11 years)	old age group (12–20 years)
*n*		677	464
Sex	*n* (%) female	323 (47.7%)	230 (49.6%)
	*n* (%) male	354 (52.3%)	234 (50.4%)
Age	mean (sd)	7.71 (2.73)	15.31 (2.32)
Parental education^a^	*n* (%) high	332 (49%)	177 (38.1%)
	*n* (%) low/ medium	345 (51%)	287 (61.9%)
BMI-SDS^b^	mean (sd)	−0.05 (0.96)	0.02 (1.19)
Weight group	*n* (%) normal weight	621 (91.7%)	403 (86.9%)
	*n* (%) overweight weight/ obesity^c^	56 (8.3%)	61 (13.1%)
NHS^d ^(range: −120 to 120)	mean (sd)	41.4 (27.28)	24.2 (27.06)
Media use during dinner	*n* (%) yes	81 (12%)	125 (26.9%)
Dinner with family	*n* (%) yes	667 (98.5%)	393 (84.7%)
Snacking	*n* (%) yes	363 (53.6%)	171 (36.9%)
Help cooking	*n* (%) yes	642 (94.8%)	440 (94.8%)
Organized PA	*n* (%) yes	398 (58.8%)	278 (59.9%)
Organized PA (h/ week)	mean (sd)	1.25 (1.65)	2.29 (3.00)
Non-organized PA	*n* (%) yes	115 (17%)	249 (53.7%)
Non-organized PA (h/week)	mean (sd)	1.08 (0.46) 261 missings	1.50 (2.28)
PA for weight loss/maintenance (range 0–10)	mean (sd)	1.94 (2.46)	4.6 (3.46)
PA enjoyment (range 0–10)	mean (sd)	8.56 (1.88)	7.71 (2.48)
Total screen time^e^	mean (sd)	1.41 h/day (1.2)	3.67 h/day (1.99)

^a^High parental education indicating a college or university degree.

^b^BMI-SDS: BMI standard deviation score (mean = 0, sd = 1).

^c^Overweight weight/ obesity: BMI-SDS > 1.282 (> 90th percentile).

^d^Nutritional health score, based on the frequency of consumption of different food groups, higher values indicate a healthier diet.

^e^Time spent with screen media (TV, smartphone, tablet, etc.) excluding schoolwork.

The young age group included 677 children (48% girls, mean age = 7.71 years, SD = 2.73). Half of the parents (49%) had a high education. On average, participants had a BMI-SDS of −0.05 (SD = 0.96). Most of the children were categorized as normal weight (n = 621, 92%), whereas 56 children were categorized as overweight/obese (8%). The average NHS was 41.4 (SD = 27.28). About 10% of the children used media during dinner, and about 50% usually snacked between meals. Nearly all had dinner with their family and helped their parents with the cooking. More than half of the children of the children participated in organized PA and 17% engaged in non-organized PA. On average, the children participated 1.25 h per week (SD = 1.65) in organized PA and 1.08 h per week (SD = 0.46) in non-organized PA.

PA for weight loss/maintenance was rated 1.94 (SD = 2.46) on average, indicating a low level of agreement. PA enjoyment was rated 8.56 (SD = 1.88), indicating a high level of agreement. The participants spent 1.41 h a day on average (SD = 1.2) using screen media, excluding schoolwork.

The old age group included 464 children and adolescents (50% girls, mean age = 15.31 years, SD = 2.32). About 40% of the parents had a high education. Participants had a BMI-SDS of 0.02 (SD = 1.19) on average. Most of the children and adolescents were categorized as normal weight (*n* = 403, 87%), whereas 61 of them were categorized as overweight/obese (13%). The average NHS was 24.2 (SD = 27.06). A quarter of the participants used media during dinner, 85% had dinner with their families, 37% usually snacked between meals and 95% helped their parents with the cooking. More than half of the children and adolescents participated in organized PA (60%) and/or non-organized PA (54%). On average, the children and adolescents participated 2.29 h per week (SD = 3.00) in organized PA and 1.50 h per week (SD = 2.28) in non-organized PA. The average rating of PA for weight loss/maintenance was 4.6 (SD = 3.46), indicating a lower level of agreement. PA enjoyment was rated 7.71 (SD = 2.48), indicating a relatively high level of agreement. The participants spent 3.67 h a day on average (SD = 1.99) using screen media, excluding schoolwork.

### BMI-SDS in relation to the independent variables

Associations between BMI-SDS and the independent variables are shown in Table 2.

**Table 2:** Associations between BMI-SDS and parental education, characteristics of diet, culture of eating, PA, and total screen time in the young and the old age group.

	young age group (2–11 years)		old age group (12–20 years)	
Independent Variables (Coefficients)	BMI-SDS Beta (95% CI)	*p*	BMI-SDS Beta (95% CI)	*p*
Parental education (high)	−0.25 (−0.4; −0.11)***^a,b^	< 0.001	−0.27 (−0.49; −0.05)*	0.02
NHS	0 (0.00; 0.00)	0.408	0 (0; 0.01)	0.223
Media use during dinner (yes)	0.21 (−0.01; 0.44)	0.063	0.53 (0.28; 0.77)***^c^	< 0.001
Dinner with family (yes)	−0.01 (−0.61; 0.59)	0.092	−0.21 (−0.52; 0.09)	0.172
Snacking (yes)	−0.05 (−0.20; 0.09)	0.475	−0.12 (−0.34; 0.11)	0.307
Help cooking (yes)	0.17 (−0.16; 0.50)	0.301	0.20 (−0.28; 0.69)	0.413
Organized PA (yes)	−0.03 (−0.18; 0.12)	0.669	−0.08 (−0.31; 0.14)	0.453
Organized PA (h/ week)	0.01 (−0.04; 0.06)	0.705	−0.01 (−0.05; 0.02)	0.446
Non-organized PA (yes)	−0.23 (−0.43; −0.02)*	0.029	−0.06 (−0.28; 0.15)	0.571
Non-organized PA (h/week)	−0.06 (−0.15, 0.03)	0.212	−0.02 (−0.07; 0.03)	0.417
PA for weight loss/maintenance	0.15 (0.12; 0.18)***^b^	< 0.001	0.12 (0.09; 0.15)***^c^	< 0.001
PA enjoyment	−0.05 (−0.09; −0.01)*^b,c^	0.01	−0.05 (−0.09; 0)*^c^	0.038
Total screen time (h/day)	0.11 (0.02; 0.16)**^c^	0.009	0.09 (0.04; 0.15)**	0.001

^a^A significant interaction with sex indicated a stronger association in girls.

^b^A significant interaction with age indicated a stronger association in older children.

^c^A significant interaction with parental education indicated a stronger association in children from families with low/medium education.

^*^p < 0.05; **p < 0.01; ***p < 0.001.

All associations are adjusted for parental educational, age and sex.

In both the young and the old age group, BMI-SDS was significantly lower in children from families with high (compared to low/medium) education (b = −0.25 (−0.4; −0.11), *p* < 0.001, and b = −0.27 (−0.49; −0.05), *p* = 0.02, respectively). This means that participants whose parents had a lower education had a higher BMI-SDS. In the young age group, a significant interaction with sex showed that this association was stronger in girls (b = −0.29 (−0.58; −0.01), p = 0.045), and a significant interaction with age further showed that the strength of the association increased with increasing age (b = −0.07 (−0.12; −0.01) *p* = 0.016).

With respect to diet, the analyses revealed no significant association between the NHS and BMI-SDS. However, media use during dinner was significantly associated with higher BMI-SDS in the older age group (b = 0.53 (0.28; 0.77), *p* < 0.001). As indicated by a significant interaction between media use during dinner and parental education (b = 0.56 (0.03; 1.09), *p* = 0.038), this association was stronger in participants whose parents had a low/medium (compared to high) education. In both age groups, we observed no significant associations between BMI-SDS and having dinner with the family, snacking and help cooking.

**Fig. 2:**
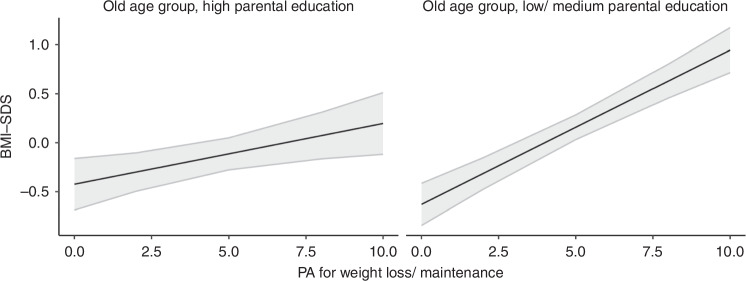
Association between BMI-SDS and PA for weight loss/maintenance in the old age group separated by parental education. Effect plots illustrating the association between BMI-SDS and PA for weight loss/maintenance ( + 95% Confidence Interval) in children and adolescents of the old age group, separated by parental education. All associations are adjusted for age and sex.

**Fig. 3:**
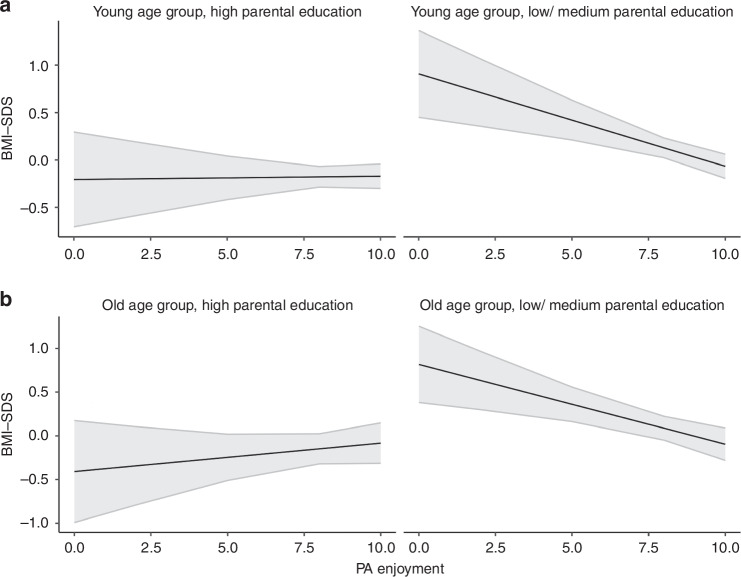
Association between BMI-SDS and PA enjoyment in both age groups separated by parental education. Effect plots illustrating the association between BMI-SDS and PA enjoyment ( + 95% Confidence Interval) in children of the young age group **a** and the old age group **b** separated by parental education. All associations are adjusted for age and sex.

Concerning PA, we observed a significant association between non-organized PA and lower BMI-SDS in the young age group only (b = −0.23 (−0.43; −0.02), *p* = 0.029). In both age groups, the analyses revealed no significant associations between organized PA and BMI-SDS. While the duration of organized and non-organized PA was not related to BMI-SDS in either age group, the participants’ motivation for being physically active was. In both age groups, PA for weight loss/maintenance was associated with higher BMI-SDS (b = 0.15 (0.12; 0.18), *p* < 0.001 and b = 0.12 (0.09; 0.15), *p* < 0.001, respectively). In the younger age group, the strength of this association increased with increasing age (b = 0.02 (0.02; 0.03), *p* < 0.001), while in the older age group, the association was stronger in families with a low/medium parental education (b = 0.1 (0.04; 0.15), *p* = 0.002, see Fig. 2). In contrast, PA enjoyment was significantly associated with lower BMI-SDS in both age groups (b = −0.05 (−0.09; −0.01), *p* = 0.01 and b = −0.05 (−0.09; 0), *p* = 0.038, respectively). Significant interactions with parental education indicated that the associations were stronger in families with low/medium parental education (b = −0.1 (−0.18; −0.02), *p* = 0.010 for the young age group (see Fig. 3a) and b = −0.12 (−0.21; −0.03), *p* = 0.007 for the old age group (see Fig. 3b)). In the young age group, a significant interaction with age (b = −0.02 (−0.03; 0.00), *p* = 0.024) further showed that the strength of the association became stronger with increasing age.

Regarding total screen time, the analyses showed significant associations with BMI-SDS in both the young age group (b = 0.11 (0.02; 0.16), *p* = 0.009) and the old age group (b = 0.09 (0.04; 0.15), *p* = 0.001). In the young age group, a significant interaction with parental education revealed that this association was stronger in children whose parents had a low/medium education (b = 0.17 (0.04; 0.29), *p* = 0.01).

In multivariate regression analyses, PA for weight loss/maintenance remained significantly associated with higher BMI-SDS in both age groups (b = 0.14, (0.12; 0.17), *p* < 0.001 in the young age group and b = 0.12 (0.09; 0.15), *p* < 0.001 in the old age group). In the young age group, high parental education was significantly associated with lower BMI-SDS (b = −0.19, (−0.32; −0.05), *p* = 0.006), and in the old age group, media use during dinner was significantly associated with higher BMI-SDS (b = 0.34, (0.1; 0.57), *p* = 0.005) in multivariate analyses.

## Discussion

The present study investigated the relationship between BMI and parental education, NHS, culture of eating (media use during dinner, having dinner with the family, snacking, help cooking), participation in and duration of organized and non-organized PA, motives for PA (PA for weight loss/maintenance, PA enjoyment), and total screen time in a large sample of children and adolescents aged 2 to 20 years from Leipzig, a city in Germany. It further examined whether these associations were moderated by parental education, age and sex.

### Main findings

This study showed that children and adolescents with a higher BMI came from families with lower formal education and spent more time using screen media. Younger children with a higher BMI participated less frequently in non-organized PA whereas older children with a higher BMI more frequently used media during dinner. Most interestingly, in both age groups, participants with a higher BMI showed less PA enjoyment. In contrast, they showed a higher motivation to be physically active to lose/maintain weight. To the best of our knowledge, this is the first study that shows that these motivational aspects are directly linked to the weight status of children and adolescents.

### Study sample and association between BMI-SDS and SES

In the current study, 8.3% of the younger children and 13.1% of the older children were overweight or obese. These rates are lower than in a German reference population.^3^ This might be explained by the underrepresentation of families with low SES.^18^ As in many cohort studies, the education of the participating families was rather high. In the annual population survey conducted by official statistics in Germany in 2019, 18.5% of people aged 20 and older had a university degree.^52^ In the present study, in contrast, nearly half of the parents in the young age group (49%) and 38.1% of the parents in the old age group had a university degree.

In line with previous studies,^17^ we showed a negative association between parental education (as indicator of SES) and BMI. In the young age group, the association was stronger in girls and older children. In the multivariate analysis, low/medium parental education remained significantly associated with higher BMI in the young age group. This association between lower SES and a higher BMI might be explained by underlying behavioral factors, e.g., unhealthier diet, less PA, and more sedentary behavior in families with a lower SES.^16,49,53,54^

The association between individual SES and BMI has been shown to depend on neighborhood SES and school SES.^55,56^ This suggests that our results could vary between different neighborhoods and schools. We were unable to adjust for different contexts because information on participants’ addresses and schools was not available.

### Association between BMI-SDS and diet

The average NHS was high in both age groups, indicating that the participants had a healthy diet. In contrast to previous findings,^19^ we found no association between BMI and nutritional health. Nutritional health was assessed via questionnaire. Therefore, we cannot rule out that certain groups of participants (e.g. those with a higher BMI) tended to answer in a more socially desirable way or were not able to correctly remember their food consumption. Previous studies showed that children with overweight/obesity tended to underreport their energy intake.^57,58^

Furthermore, evidence suggests that FFQs have limitations in accuracy among children and adolescents.^59^ Including questions about eating behavior and context might provide a more comprehensive and accurate assessment of dietary habits. This approach is consistent with the American Academy of Pediatrics’ emphasis on considering cultural and behavioral factors in dietary assessment.^60^

Only 12% of the younger children used media during dinner, whereas more than a quarter of the older children did so. That is in line with previous findings that show an increased use of media while eating with increasing child age.^49^ Participants in the old age group who used media during dinner had a significantly higher BMI, and this association remained significant in the multivariate analysis. The link between media use during dinner and higher BMI was stronger in participants whose parents had low/medium education. O’Connor et al. found no association between TV watching while eating and BMI-z-scores in preschool-aged children from low income families.^22^ The association between media use during dinner and higher BMI may only become apparent at an older age, when using media during mealtimes becomes more frequent. Then, eating in front of a screen might distract from a feeling of fullness or from the memory of the amount eaten and, therefore, lead to overconsumption.^22^

More than half of the younger children (53.6%) and more than a third of the older children (36.9%) reported to snack between mealtimes. We found no association between BMI and snacking. A reason for that may be that snacking contributes to energy needs and causes satiety,^27^ so that children and adolescents might eat less during main meals. Almost all children and adolescents of both age groups (94.8% in both groups) helped their parents with cooking. Similarly, the frequency of eating together as a family was very high (99% in the younger, 85% in the older age group). Although studies show that family dinners and helping with meal preparation are associated with a healthier diet,^24,25,29,57^ we found no association with BMI in either age group. This might be due to the low frequency of children not having family dinners or not helping their parents with cooking.

### Association between BMI-SDS and PA

In both age groups, approximately 60% participated in organized sports. Only 17% of the younger children but more than half of the older children and adolescents participated in non-organized sports.

Both younger and older participants showed high levels of PA enjoyment. PA for weight loss/maintenance was rated low by the younger age group and moderately low by the older age group. In line with previous findings,^61^ younger children who participated in non-organized PA had a lower BMI. Other than expected, there was no association between BMI and participation in organized PA. Additionally, time spent per week in organized or unorganized PA was not associated with BMI in either age group. This is in contrast to other findings,^30–32^ and could be explained by the subjective nature of the measures. Participation in and duration of PA were determined by self- and parent-reports and could be subject to biases such as social desirability and recall bias.

Participants with a higher BMI were more likely to be motivated to exercise by weight loss reasons and less likely to enjoy PA. Although a comparable pattern was already shown in adults,^39^ this is the first study that shows this phenomenon in children. Previous studies show that adolescents who enjoy PA spend more time exercising^36^ and that there is a relationship between PA enjoyment and lower BMI, mediated by segmented PA in adolescents.^35^ This suggests that children and adolescents who enjoy PA may spend more time exercising and therefore have a lower BMI. A possible reason why children with higher BMI enjoy PA less is a negative body image. A previous study suggests that youth with obesity have a more negative body image,^62^ which is associated with reduced PA engagement.^63^ Also, adolescents with overweight or obesity might be less likely to participate in PA due to psychological stress, such as weight-based bullying and stigmatization, which can reinforce negative stereotypes and self-perceptions and, therefore, further diminish their enjoyment of activity.^64,65^

Our analyses show that the association between lower BMI and PA enjoyment was stronger in less educated families in both age groups. The fact that intrinsic motivation for PA has a protective effect on weight for children from low-SES families in particular may be due to the lack of other incentives like parental encouragement or social support: Parents with a lower SES were shown to be less likely to encourage their children to participate in PA.^66^ Children and adolescents in their direct environment might be less physically active as studies show that SES is inversely related to PA and sports participation in youth.^53^

In the young age group, the strength of the association between BMI and PA enjoyment further increased with increasing age. The association between higher BMI and PA for weight loss/maintenance was stronger in families with a lower education in the old age group whereas in the young age group the strength of this association increased with increasing age. In both age groups, PA for weight loss/maintenance remained significantly associated with higher BMI in multivariate analyses.

These findings could implicate that public health communication about the health benefits of PA may not be achieving its intended objective. Rather than encouraging PA for health and therefore fostering extrinsic motives, it may be more effective to implement strategies that foster the enjoyment of PA, with the long-term goal of improving health due to increased PA levels.

It should be noted that to determine intrinsic and extrinsic motivation for PA only two questions were examined. Other intrinsic motivating factors like a sense of self-efficacy^35^ and extrinsic motives like family-^66^ or peer-support^35^ and parental- and peer-norms^67^ have been shown to be linked to PA participation. It would be interesting to investigate whether these and other motivating factors, such as noting increased skills, the feeling of achievement, stress-relief or -distraction are associated with the weight status of children and adolescents.

### Association between BMI-SDS and screen time

The average screen time in this study was 1.41 h/day in the young age group and 3.67 h/day in the old age group. It is recommended that children from 5 to 17 years spend less than 2 h/day using screen media.^41^ Thus, average screen time exceeded the recommended limit in the old age group. This finding is consistent with recent studies showing that the use of screen media plays an increasingly important role in the life of children and adolescents.^40^

In line with our hypothesis, participants with a higher BMI spent significantly more time using screen media in both age groups. For younger children, the strength of that association was stronger in families with a low/medium education. In these families, the impact of high media use may be particularly strong because there are fewer other resources available to compensate for this effect. At the same time, children with a higher BMI from low SES families may be especially interested in media use because alternative activities are less accessible.^68^ Many studies show that screen time is associated with a higher BMI.^43,44^ There is also consistent evidence that children with low SES have even higher screen time.^20,40^

### Strengths and limitations

The investigation of various behavioral factors and motives and their association with BMI-SDS in a large sample of children and adolescents with a wide age range is a strength of this study. We also considered interactions with sex, age, and parental education. However, the underrepresentation of families with low education (low SES) limits the generalizability of our findings.^69^ Neighborhood and school SES were not considered, which limits the findings of this study, as the association between BMI and SES might be context dependent.

The data were based on self-/parent-reporting, which can lead to biases such as social desirability and recall bias. Self-reporting of participation and duration of organized and non-organized PA without information on intensity or type of activity limits the validity of the measure. Energy expenditure and potential weight gain are influenced by the intensity of PA, which is better measured by more specific questionnaires or accelerometers. Intrinsic and extrinsic motivation was assessed with one question each and other motivating factors were not considered. Further, parents may not be able to accurately assess their children’s motives participating in PA. In addition, the parents’ answers for their children and those of the children themselves may differ, which limits the comparability of the results of the younger and older age samples. A further limitation of the study is the long time period, as the data used covers a two-year interval. Finally, the cross-sectional design of the study doesn’t allow for causal conclusions. It is not possible to determine the direction of relationships between the variables. While the study can identify associations, it cannot establish cause-and-effect relationships.

## Conclusion

A lower weight in children and adolescents is linked with high parental education, screen-free eating, higher participation in non-organized PA and less screen-time. While measures of motivation were limited in this study, our findings suggest that intrinsic motivation for PA is associated with lower weight whereas extrinsic motivation for PA is associated with higher weight. Future studies should examine whether public health strategies aimed at influencing these factors could have a positive effect on the BMI in children and adolescents. More robust measures of intrinsic and extrinsic motivation should be investigated.

## Supplementary information

Supplementary information

## Data Availability

Data collected in the LIFE Child study are not publicly available, as the publication of data is not covered by the informed consent provided by study participants. Because datasets contain potentially sensitive information, all researchers intending to access data are required to sign a project agreement. Researchers interested in accessing and analyzing data from the LIFE Child study may contact the data use and access committee (forschungsdaten@medizin.uni-leipzig.de).

## References

[1] GBD 2015 Obesity Collaborators. Health effects of overweight and obesity in 195 countries over 25 years. *N. Engl. J. Med.* **377**, 13–27 (2017). 28604169 10.1056/NEJMoa1614362 PMC5477817

[2] Wabitsch, M., Moss, A. & Kromeyer-Hauschild, K. Unexpected plateauing of childhood obesity rates in developed countries. *BMC Med.* **12**, 17 (2014). 24485015 10.1186/1741-7015-12-17 PMC3908468

[3] Schienkiewitz, A., Damerow, S., Schaffrath Rosario, A. & Kurth, B.-M. Body-Mass-Index von Kindern und Jugendlichen: Prävalenzen und Verteilung unter Berücksichtigung von Untergewicht und extremer Adipositas. *Bundesgesundheitsblatt - Gesundheitsforschung - Gesundheitsschutz* **62**, 1225–1234 (2019). 31529189 10.1007/s00103-019-03015-8

[4] Vogel, M. et al. Age- and weight group-specific weight gain patterns in children and adolescents during the 15 years before and during the COVID-19 pandemic. *Int. J. Obes.* **46**, 144–152 (2022). 10.1038/s41366-021-00968-2 PMC8458556 34556774

[5] Lindberg, L., Hagman, E., Danielsson, P., Marcus, C. & Persson, M. Anxiety and depression in children and adolescents with obesity: a nationwide study in Sweden. *BMC Med* **18**, 30 (2020). 32079538 10.1186/s12916-020-1498-z PMC7033939

[6] Fang, X. et al. Childhood obesity leads to adult type 2 diabetes and coronary artery diseases: A 2-sample mendelian randomization study. *Medicine* **98**, e16825 (2019). 31393416 10.1097/MD.0000000000016825 PMC6708873

[7] Jensen, B. W., Meyle, K. D., Madsen, K., Sørensen, T. I. A. & Baker, J. L. Early life body size in relation to risk of renal cell carcinoma in adulthood: a Danish observational cohort study. *Eur. J. Epidemiol.* **35**, 251–258 (2020). 31993884 10.1007/s10654-020-00605-8

[8] Horesh, A., Tsur, A. M., Bardugo, A. & Twig, G. Adolescent and childhood obesity and excess morbidity and mortality in young adulthood—a systematic review. *Curr. Obes. Rep.* **10**, 301–310 (2021). 33950400 10.1007/s13679-021-00439-9

[9] Ward, Z. J. et al. Simulation of growth trajectories of childhood obesity into adulthood. *N. Engl. J. Med.* **377**, 2145–2153 (2017). 29171811 10.1056/NEJMoa1703860 PMC9036858

[10] Malhotra, S., Sivasubramanian, R. & Singhal, V. Adult obesity and its complications: a pediatric disease? *Curr. Opin. Endocrinol. Diabetes Obes.* **28**, 46–54 (2021). 33229926 10.1097/MED.0000000000000592

[11] Yang, J. et al. The impact of age-specific childhood body-mass index on adult cardiometabolic traits: a Mendelian randomization study. *Front. Endocrinol.* **14**, 1159547 (2024). 10.3389/fendo.2023.1159547 PMC10822942 38288476

[12] Zhang, T. et al. Trajectories of childhood BMI and adult diabetes: the Bogalusa Heart Study. *Diabetologia* **62**, 70–77 (2019). 30343393 10.1007/s00125-018-4753-5 PMC6365010

[13] Mcleroy, K., Bibeau, D., Steckler, A. & Glanz, K. An ecology perspective on health promotion programs. *Health Educ. Q.* **15**, 351–377 (1988). 3068205 10.1177/109019818801500401

[14] Jernigan, J. et al. Childhood obesity declines project: highlights of community strategies and policies. *Child. Obes.* **14**, S-32–S-39 (2018). 29565654 10.1089/chi.2018.0022 PMC5865627

[15] Jebeile, H., Kelly, A. S., O’Malley, G. & Baur, L. A. Obesity in children and adolescents: epidemiology, causes, assessment, and management. *Lancet Diabetes Endocrinol.* **10**, 351–365 (2022). 35248172 10.1016/S2213-8587(22)00047-X PMC9831747

[16] Poulain, T. et al. Associations between socio-economic status and child health: findings of a large German cohort study. *Int. J. Environ. Res. Public. Health* **16**, 677 (2019). 30813530 10.3390/ijerph16050677 PMC6427670

[17] Barriuso, L. *et al*. Socioeconomic position and childhood-adolescent weight status in rich countries: a systematic review, 1990–2013. *BMC Pediatr*. **15**, (2015). 10.1186/s12887-015-0443-3 PMC4578240 26391227

[18] Schienkiewitz, A., Brettschneider, A. K., Damerow, S. & Rosario, A. S. Übergewicht und Adipositas im Kindes- und Jugendalter in Deutschland – Querschnittergebnisse aus KiGGS Welle 2 und Trends. 10.17886/RKI-GBE-2018-005.2 (2018).

[19] Mahumud, R. A. et al. Association of dietary intake, physical activity, and sedentary behaviours with overweight and obesity among 282,213 adolescents in 89 low and middle income to high-income countries. *Int. J. Obes.* **45**, 2404–2418 (2021). 10.1038/s41366-021-00908-0 34274952

[20] Avery, A., Anderson, C. & McCullough, F. Associations between children’s diet quality and watching television during meal or snack consumption: A systematic review. *Matern. Child. Nutr.* **13**, e12428 (2017). 28211230 10.1111/mcn.12428 PMC6866147

[21] Magriplis, E., Farajian, P., Panagiotakos, D. B., Risvas, G. & Zampelas, A. The relationship between behavioral factors, weight status and a dietary pattern in primary school aged children: The GRECO study. *Clin. Nutr.* **38**, 310–316 (2019). 29398340 10.1016/j.clnu.2018.01.015

[22] O’Connor, T. M. et al. The association of TV viewing during dinner meals with quality of dietary intake and BMI z-scores among low income, ethnic minority preschool children. *Appetite* **140**, 231–238 (2019). 31121200 10.1016/j.appet.2019.05.023

[23] Rodrigues, L. et al. Taste sensitivity and lifestyle are associated with food preferences and BMI in children. *Int. J. Food Sci. Nutr.* **71**, 875–883 (2020). 32188327 10.1080/09637486.2020.1738354

[24] Fulkerson, J. A., Larson, N., Horning, M. & Neumark-Sztainer, D. A review of associations between family or shared meal frequency and dietary and weight status outcomes across the lifespan. *J. Nutr. Educ. Behav.* **46**, 2–19 (2014). 24054888 10.1016/j.jneb.2013.07.012

[25] Lee, J. et al. Are patterns of family evening meal practices associated with child and parent diet quality and weight-related outcomes? *Appetite* **171**, 105937 (2022). 35045323 10.1016/j.appet.2022.105937 PMC8892840

[26] Bellisle, F. Meals and snacking, diet quality and energy balance. *Physiol. Behav.* **134**, 38–43 (2014). 24657181 10.1016/j.physbeh.2014.03.010

[27] Njike, V. Y. et al. Snack food, satiety, and weight. *Adv. Nutr. Bethesda Md.* **7**, 866–878 (2016). 10.3945/an.115.009340 PMC5015032 27633103

[28] Larson, N. I., Miller, J. M., Watts, A. W., Story, M. T. & Neumark-Sztainer, D. R. Adolescent snacking behaviors are associated with dietary intake and weight status. *J. Nutr.* **146**, 1348–1355 (2016). 27281807 10.3945/jn.116.230334 PMC4926852

[29] Ng, C. M., Kaur, S., Koo, H. C. & Mukhtar, F. Involvement of children in hands-on meal preparation and the associated nutrition outcomes: A scoping review. *J. Hum. Nutr. Diet.* **35**, 350–362 (2022). 33938062 10.1111/jhn.12911

[30] Griffiths, L. J. et al. Objectively measured physical activity and sedentary time: cross-sectional and prospective associations with adiposity in the Millennium Cohort Study. *BMJ Open* **6**, e010366 (2016). 27067891 10.1136/bmjopen-2015-010366 PMC4838720

[31] Dowda, M., Taverno Ross, S. E., McIver, K. L., Dishman, R. K. & Pate, R. R. Physical activity and changes in adiposity in the transition from elementary to middle school. *Child. Obes.* **13**, 53–62 (2017). 27929670 10.1089/chi.2016.0103 PMC5278816

[32] Dowda, M., Saunders, R. P., Dishman, R. K. & Pate, R. R. Association of physical activity, sedentary behavior, diet quality with adiposity: a longitudinal analysis in children categorized by baseline weight status. *Int. J. Obes.* **48**, 240–246 (2024). 10.1038/s41366-023-01405-2 37932409

[33] Ryan, R. M. & Deci, E. L. Intrinsic and extrinsic motivations: classic definitions and new directions. *Contemp. Educ. Psychol.* **25**, 54–67 (2000). 10620381 10.1006/ceps.1999.1020

[34] Sebire, S. J., Standage, M. & Vansteenkiste, M. Examining intrinsic versus extrinsic exercise goals: cognitive, affective, and behavioral outcomes. *J. Sport Exerc. Psychol.* **31**, 189–210 (2009). 19454771 10.1123/jsep.31.2.189

[35] Fu, Y., Burns, R. D., Hsu, Y.-W. & Zhang, P. Motivation, segmented physical activity, sedentary behavior, and weight status in adolescents: A path analysis. *Res. Q. Exerc. Sport* **93**, 204–209 (2022). 32897846 10.1080/02701367.2020.1804520

[36] Michael, S. L., Coffield, E., Lee, S. M. & Fulton, J. E. Variety, enjoyment, and physical activity participation among high school students. *J. Phys. Act. Health* **13**, 223–230 (2016). 26107142 10.1123/jpah.2014-0551 PMC5295133

[37] Haas, P., Yang, C.-H. & Dunton, G. F. Associations between physical activity enjoyment and age-related decline in physical activity in children—results from a longitudinal within-person study. *J. Sport Exerc. Psychol.* **43**, 205–214 (2021). 33811189 10.1123/jsep.2020-0156

[38] Pannekoek, L., Piek, J. P. & Hagger, M. S. Motivation for physical activity in children: A moving matter in need for study. *Hum. Mov. Sci.* **32**, 1097–1115 (2013). 24100193 10.1016/j.humov.2013.08.004

[39] Anić, P., Pokrajac-Bulian, A. & Mohorić, T. Role of sociocultural pressures and internalization of appearance ideals in the motivation for exercise. *Psychol. Rep.* **125**, 1628–1647 (2022). 33752514 10.1177/00332941211000659

[40] Auhuber, L., Vogel, M., Grafe, N., Kiess, W. & Poulain, T. Leisure activities of healthy children and adolescents. *Int. J. Environ. Res. Public. Health* **16**, 2078 (2019). 31212786 10.3390/ijerph16122078 PMC6617342

[41] Tremblay, M. S. et al. Canadian 24-hour movement guidelines for children and youth: An integration of physical activity, sedentary behaviour, and sleep. *Appl. Physiol. Nutr. Metab. Physiol. Appl. Nutr. Metab.* **41**, S311–S327 (2016). 10.1139/apnm-2016-0151 27306437

[42] Council on Communications and Media. et al. Media and young minds. *Pediatrics* **138**, e20162591 (2016). 27940793 10.1542/peds.2016-2591

[43] Schwarzfischer, P. et al. Effects of screen time and playing outside on anthropometric measures in preschool aged children. *PLoS ONE* **15**, e0229708 (2020). 32119714 10.1371/journal.pone.0229708 PMC7051070

[44] Nagata, J. M. et al. Association of physical activity and screen time with body mass index among US adolescents. *JAMA Netw. Open* **6**, e2255466 (2023). 36757695 10.1001/jamanetworkopen.2022.55466 PMC9912127

[45] Poulain, T. et al. The LIFE Child study: a population-based perinatal and pediatric cohort in Germany. *Eur. J. Epidemiol.* **32**, 145–158 (2017). 28144813 10.1007/s10654-016-0216-9

[46] Quante, M. et al. The LIFE child study: a life course approach to disease and health. *BMC Public Health* **12**, 1021 (2012). 23181778 10.1186/1471-2458-12-1021 PMC3533937

[47] Kromeyer-Hauschild, K. et al. Perzentile für den Body-mass-Index für das Kindes- und Jugendalter unter Heranziehung verschiedener deutscher Stichproben. *Monatsschr. Kinderheilkd.* **149**, 807–818 (2001).

[48] Lampert, T., Hoebel, J., Kuntz, B., Müters, S. & Kroll, L. E. Messung des sozioökonomischen Status und des subjektiven sozialen Status in KiGGS Welle 2. 10.25646/2968 (2018).

[49] Poulain, T., Spielau, U., Vogel, M., Körner, A. & Kiess, W. CoCu: A new short questionnaire to evaluate diet composition and culture of eating in children and adolescents. *Clin. Nutr.* **38**, 2858–2865 (2019). 30616881 10.1016/j.clnu.2018.12.020

[50] Stiegler, P. et al. A new FFQ designed to measure the intake of fatty acids and antioxidants in children. *Public Health Nutr.* **13**, 38–46 (2010). 19476676 10.1017/S1368980009005813

[51] Kersting, M., Alexy, U. & Clausen, K. Using the concept of food based dietary guidelines to develop an Optimized Mixed Diet (OMD) for German children and adolescents. *J. Pediatr. Gastroenterol. Nutr.* **40**, 301–308 (2005). 15735483 10.1097/01.mpg.0000153887.19429.70

[52] Statistisches Bundesamt Deutschland - GENESIS-Online. https://www-genesis.destatis.de/genesis/online?operation=abruftabelleBearbeiten&levelindex=0&levelid=1723478153419&auswahloperation=abruftabelleAuspraegungAuswaehlen&auswahlverzeichnis=ordnungsstruktur&auswahlziel=werteabruf&code=12211-9013&auswahltext=&werteabruf=Werteabruf#abreadcrumb (2024).

[53] Tandon, P. S. et al. Socioeconomic inequities in youth participation in physical activity and sports. *Int. J. Environ. Res. Public. Health* **18**, 6946 (2021). 34209544 10.3390/ijerph18136946 PMC8297079

[54] Krist, L. et al. Association of individual and neighbourhood socioeconomic status with physical activity and screen time in seventh-grade boys and girls in Berlin, Germany: a cross-sectional study. *BMJ Open* **7**, e017974 (2017). 29288179 10.1136/bmjopen-2017-017974 PMC5770905

[55] Luiggi, M., Rey, O., Travert, M. & Griffet, J. Overweight and obesity by school socioeconomic composition and adolescent socioeconomic status: a school-based study. *BMC Public Health* **21**, 1837 (2021). 34635065 10.1186/s12889-021-11752-2 PMC8507235

[56] Kim, Y., Landgraf, A. & Colabianchi, N. Living in high-SES neighborhoods is protective against obesity among higher-income children but not low-income children: Results from the healthy communities study. *J. Urban Health* **97**, 175–190 (2020). 32107723 10.1007/s11524-020-00427-9 PMC7101452

[57] Gomes, D. et al. A simple method for identification of misreporting of energy intake from infancy to school age: Results from a longitudinal study. *Clin. Nutr.* **37**, 1053–1060 (2018). 28780991 10.1016/j.clnu.2017.05.003

[58] Jessri, M., Lou, W. Y. & L’Abbé, M. R. Evaluation of different methods to handle misreporting in obesity research: evidence from the Canadian national nutrition survey. *Br. J. Nutr.* **115**, 147–159 (2016). 26522666 10.1017/S0007114515004237

[59] Saravia, L. et al. Relative validity of FFQ to assess food items, energy, macronutrient and micronutrient intake in children and adolescents: a systematic review with meta-analysis. *Br. J. Nutr.* **125**, 792–818 (2021). 32807247 10.1017/S0007114520003220

[60] Hampl, S. E. et al. Clinical practice guideline for the evaluation and treatment of children and adolescents with obesity. *Pediatrics* **151**, e2022060640 (2023). 36622115 10.1542/peds.2022-060640

[61] Schwarzfischer, P. et al. Longitudinal analysis of physical activity, sedentary behaviour and anthropometric measures from ages 6 to 11 years. *Int. J. Behav. Nutr. Phys. Act.* **15**, 126 (2018). 30526600 10.1186/s12966-018-0756-3 PMC6286599

[62] Moehlecke, M., Blume, C. A., Cureau, F. V., Kieling, C. & Schaan, B. D. Self-perceived body image, dissatisfaction with body weight and nutritional status of Brazilian adolescents: a nationwide study. *J. Pediatr.* **96**, 76–83 (2020). 10.1016/j.jped.2018.07.006 PMC9432337 30098939

[63] Gualdi-Russo, E., Rinaldo, N. & Zaccagni, L. Physical activity and body image perception in adolescents: A systematic review. *Int. J. Environ. Res. Public. Health* **19**, 13190 (2022). 36293770 10.3390/ijerph192013190 PMC9603811

[64] Ajibewa, T. A. et al. Psychological stress and lowered physical activity enjoyment in adolescents with overweight/obesity. *Am. J. Health Promot.* **35**, 766–774 (2021). 33626891 10.1177/0890117121997042

[65] Slater, A. & Tiggemann, M. Gender differences in adolescent sport participation, teasing, self-objectification and body image concerns. *J. Adolesc.* **34**, 455–463 (2011). 20643477 10.1016/j.adolescence.2010.06.007

[66] George, A. M. et al. Association between socio-economic status and physical activity is mediated by social support in Brazilian students. *J. Sports Sci.* **37**, 500–506 (2019). 30124376 10.1080/02640414.2018.1509435

[67] Rice, E. L. & Klein, W. M. P. Interactions among perceived norms and attitudes about health-related behaviors in U.S. adolescents. *Health Psychol.* **38**, 268–275 (2019). 30762406 10.1037/hea0000722 PMC6423530

[68] Tandon, P. S. et al. Home environment relationships with children’s physical activity, sedentary time, and screen time by socioeconomic status. *Int. J. Behav. Nutr. Phys. Act.* **9**, 88 (2012). 22835155 10.1186/1479-5868-9-88 PMC3413573

[69] Lampert, T., Müters, S., Stolzenberg, H., Kroll, L. E. & KiGGS Study Group. Messung des sozioökonomischen Status in der KiGGS-Studie. *Bundesgesundheitsblatt - Gesundheitsforschung - Gesundheitsschutz* **57**, 762–770 (2014). 24950825 10.1007/s00103-014-1974-8

