# Supplementary material for: Associations of behavioral, motivational, and socioeconomic factors with BMI among children and adolescents
Source: Pediatr Res. 2025 Jan 17;98(2):532–40. doi: 10.1038/s41390-025-03860-1 (PMC12454132; doi:10.1038/s41390-025-03860-1)
Supplement: Supplementary file 1 — Supplementary information [file 41390_2025_3860_MOESM1_ESM.docx]

**Supplementary information**

**Article:** Associations of behavioral, motivational, and socioeconomic factors with BMI among children and adolescents

**Authors:** Charlotte Jungehuelsing, Christof Meigen, Sarah Krause, Wieland Kiess, Tanja Poulain

**Table 1. Questionnaire used for the assessment of parental education**

| Question | German original^a^ | English translation |
| --- | --- | --- |
| **highest general school-leaving qualification** | Noch keinen Abschluss (noch Schüler) | No qualification yet (still a student) |
|  | Abschluss nach höchstens 7 Jahren Schulbesuch | Graduation after a maximum of 7 years of schooling |
|  | Haupt-/Volksschule | Elementary school leaving certificate/ lower secondary school leaving certificate |
|  | Realschule/Mittlere Reife/Mittlerer Schulabschluss (MSA)/Polytechnische Oberschule (POS) | Intermediate school certificate/ middle school leaving certificate/ polytechnic secondary school |
|  | Abitur, allgemeine oder fachgebundene Hochschulreife, erweiterte Oberschule (EOS), Fachhochschulreife/Fachoberschule | Higher education entrance qualification/ subject-linked university entrance qualification/ extended secondary school/ advanced technical certificate/ technical secondary school |
|  | Anderer Schulabschluss (z. B. im Ausland erworben) | Other school-leaving certificate (e.g. obtained abroad) |
| **highest professional qualification** | Keinen Abschluss, noch in beruflicher Ausbildung, z. B. Student/in, AZUBI, Berufsvorbereitungsjahr, Praktikant/in | No degree, still in professional training, e.g. student, trainee, vocational preparation year, intern |
|  | Keinen Berufsabschluss und nicht in Ausbildung | No professional qualification and not in training |
|  | Lehre, also beruflich-betriebliche Ausbildung | Apprenticeship, i.e. vocational and in-company training |
|  | Ausbildung an Berufsfachschule, Handelsschule, also beruflich-schulische Ausbildung | Training at vocational school, commercial school, i.e. vocational-school training |
|  | Fachschule, z. B. Meister-, Technikerschule, Berufs- oder Fachakademie | Technical school, e.g. master craftsman school, technical school, vocational or technical academy |
|  | Fachhochschule, Ingenieurschule | University of applied sciences, engineering school |
|  | Universität oder Hochschule | University or college |
|  | Anderen Ausbildungsabschluss (z. B. im Ausland erworben) | Other educational qualification (e.g. obtained abroad) |

^a^ Lampert, T., Hoebel, J., Kuntz, B., Müters, S. & Kroll, L. E. Messung des sozioökonomischen Status und des subjektiven sozialen Status in KiGGS Welle 2. (2018) doi:10.25646/2968.
